# Expression and Clinical Significance of the Novel Long Noncoding RNA ZNF674-AS1 in Human Hepatocellular Carcinoma

**DOI:** 10.1155/2016/3608914

**Published:** 2016-11-08

**Authors:** Lufei Zhang, Tianyu He, Yingcai Yan, Yuan Zhang, Xiaohu Zhou, Pengfei Huang, Yang Kong, Minjie Xie, Linshi Zhang, Qiang Sun, Dongkai Zhou, Haiyang Xie, Lin Zhou, Shusen Zheng, Weilin Wang

**Affiliations:** ^1^Division of Hepatobiliary and Pancreatic Surgery, Department of Surgery First Affiliated Hospital, School of Medicine, Zhejiang University, Hangzhou 310003, China; ^2^Key Laboratory of Combined Multiorgan Transplantation, Ministry of Public Health, Key Laboratory of Organ Transplantation, Zhejiang Province, Hangzhou 310003, China; ^3^Collaborative Innovation Center for Diagnosis and Treatment of Infectious Diseases, The First Affiliated Hospital, College of Medicine, Zhejiang University, Hangzhou 310003, China

## Abstract

Long noncoding RNAs (lncRNAs) play crucial roles in cancer occurrence and progression. However, the relationship between the expression levels of lncRNAs and the hepatocellular carcinoma (HCC) process is unclear. The goal of this study was to determine the expression level of ZNF674-AS1, a newly found lncRNA, in HCC and its clinical association. The expression of ZNF674-AS1 in 137 pairs of tumorous and adjacent normal tissues from patients with HCC was detected by quantitative real-time reverse transcription polymerase chain reaction. Additionally, the potential associations between its level in HCC tissue and clinicopathological features were analyzed. The expression of ZNF674-AS1 in the HCC cell lines HepG2, HCCLM3, SK-Hep1, HuH7, Hep3B, and MHCC97H was significantly downregulated compared with that in the normal liver cell line QSG-7701. The expression of ZNF674-AS1 was downregulated in 72% (99/137) of HCC tissues compared with that in paired adjacent normal tissues (*p* < 0.01). The results showed that the ZNF674-AS1 expression level was significantly correlated with metastasis (*p* = 0.041), clinical stage (*p* = 0.039), and histopathologic grading (*p* = 0.045). In addition, the Kaplan–Meier survival curves revealed that low ZNF674-AS1 expression was associated with poor prognosis in patients with HCC. Our data suggest that ZNF674-AS1 may play some role during cancer occurrence and progression and may be a new biomarker for HCC.

## 1. Introduction

Hepatocellular carcinoma (HCC) is a leading cause of cancer death in many Asian and African countries [1]. HCC causes approximately 662,000 deaths each year worldwide and about half of them occur in China [2]. The major causes of HCC are viral infections and alcohol and tobacco use.

Intensive investigations over the last few decades have focused on the role of protein-coding genes in the pathogenesis of HCC, and efforts have been made to identify appropriate prognostic markers for HCC [3–6], including primary tumor size, elevated alpha-fetoprotein levels, and gene expression markers in the primary tumor. However, these methods have not proven to be adequate in predicting the prognosis of all patients with HCC. In addition, recent studies have indicated that several long noncoding RNAs (lncRNAs) are dysregulated in HCC, and their aberrant expression levels are associated with tumorigenesis, metastasis, prognosis, or diagnosis [7–12].

lncRNAs are non-protein-coding transcripts longer than 200 nucleotides that lack an open reading frame of significant length [13–17]. As a new type of regulatory RNA molecule, lncRNA has a diverse subcellular location and plays important roles in many aspects of cell activity. Further, lncRNAs regulate gene expression at the epigenetic, transcriptional, posttranscriptional, and translational levels during cancer development [13, 18]. ZNF674 antisense RNA 1 (ZNF674-AS1, NR_015378) is an lncRNA that was first identified from the lncRNA expression profile of HCC identified by microarray analysis [10]. However, its association with HCC is unclear. The goal of the present study was to determine the expression level of ZNF674-AS1 in HCC and then to evaluate the relationship between its expression levels and clinical pathological characteristics of patients with HCC.

## 2. Materials and Methods

### 2.1. Patient Data and Tissue Samples

One hundred and thirty-seven fresh HCC tissue samples and matched normal adjacent tissue samples from 2010 to 2014 were selected from patients who underwent resection of primary HCC at our cancer center in the Department of Hepatobiliary and Pancreatic Surgery, The First Affiliated Hospital, College of Medicine, Zhejiang University, China. Tumor tissues and paired adjacent nontumorous tissues 5 cm from the edge of the tumor were obtained during surgery. None of the patients received preoperative therapy. The resected tumor and paired nontumor tissue specimens were immediately frozen in liquid nitrogen and kept at −80°C until analysis [12]. The diagnosis of each specimen was confirmed histopathologically. All of the clinical data were collected by physicians, and the researchers were blinded to the clinical data. The study was approved by the Human Research Ethics Committee of The First Affiliated Hospital, College of Medicine, Zhejiang University. Written informed consent was obtained from all of the subjects.

### 2.2. Total RNA Preparation and Real-Time Quantitative Reverse Transcription Polymerase Chain Reaction (qRT-PCR) Detection

Total RNA was isolated using TRIzol® Reagent (Life Technologies Corporation, Carlsbad, CA, USA) according to the manufacturer's protocol, and cDNA was synthesized (Bio-Rad, Hercules, CA, USA). Real-time PCR was performed according to the manufacturer's instructions.

The expression levels of glyceraldehyde-3-phosphate dehydrogenase (GAPDH) and ZNF674-AS1 were evaluated using real-time qRT-PCR. The primers were as follows: ZNF674-AS1 forward (5′-CAAAGCCTGTGGCCGATGTG-3′) and reverse (5′-ATGGTCACACATTCCTTCTCCC-3′) and GAPDH forward (5′-AGAAGGCTGGGGCTCATTTG-3′) and reverse (5′-AGGGGCCATCCACAGTCTTC-3′). The cDNAs were amplified using an Applied Biosystems 7500-fast PCR machine; the reaction was performed according to the PCR kit instructions. The cycling conditions were as follows: denaturation at 94°C for 5 min, followed by 40 cycles of 95°C for 30 s, 58°C for 30 s, and 72°C for 20 s. All of the experiments were conducted three times, and the average was determined. The 2^−ΔΔCt^ formula was used to calculate differential gene expression [19].

### 2.3. Cell Lines and Cell Culture

Six HCC cell lines (HepG2, HCCLM3, SK-Hep1, HuH7, Hep3B, and MHCC97H) and one normal liver cell line (QSG-7701), all of which are maintained at our institution, were used in this study. All of the cell lines were maintained in a humidified atmosphere containing 5% CO_2_ at 37°C and were passaged using standard cell culture techniques [19].

### 2.4. Statistical Analysis

The relationship between ZNF674-AS1 expression and clinicopathological variables was assessed using a *χ* ^2^ test. All of the statistical analyses were performed using SPSS 19.0 for Windows (SPSS, Chicago, IL, USA) and GraphPad Prism® 5.0 (GraphPad Software, La Jolla, CA, USA). A one-way analysis of variance (ANOVA) and Student's* t*-tests were used as appropriate. Overall survival curves were plotted according to the Kaplan–Meier method. A *p* value less than 0.05 was deemed to indicate statistical significance.

## 3. Results

### 3.1. ZNF674-AS1 Was Downregulated in HCC Cell Lines and Tissues

**Figure 1:**
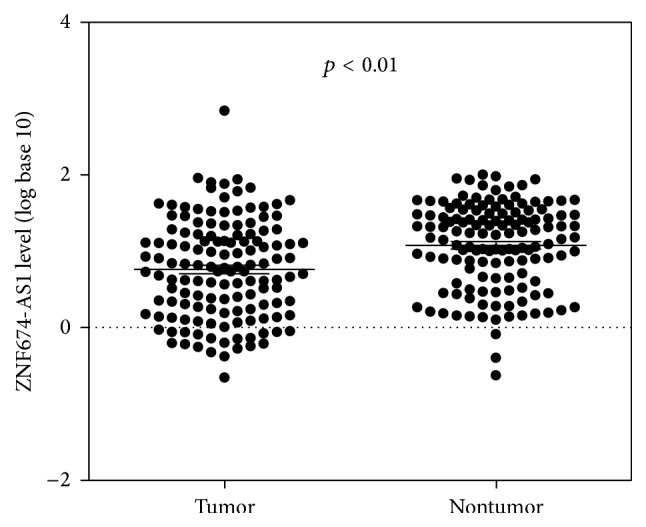
In all 137 tissue pairs, the downregulation of ZNF674-AS1 expression was significant in tumors compared to adjacent noncancerous tissues.

**Figure 2:**
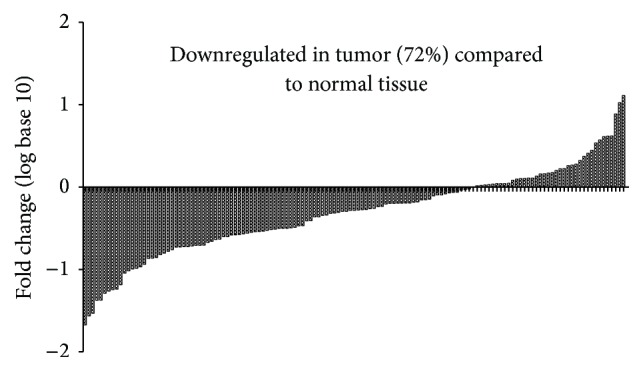
The ZNF674-AS1 expression levels in hepatocellular carcinoma (HCC) tissues was reduced (72%).

**Figure 3:**
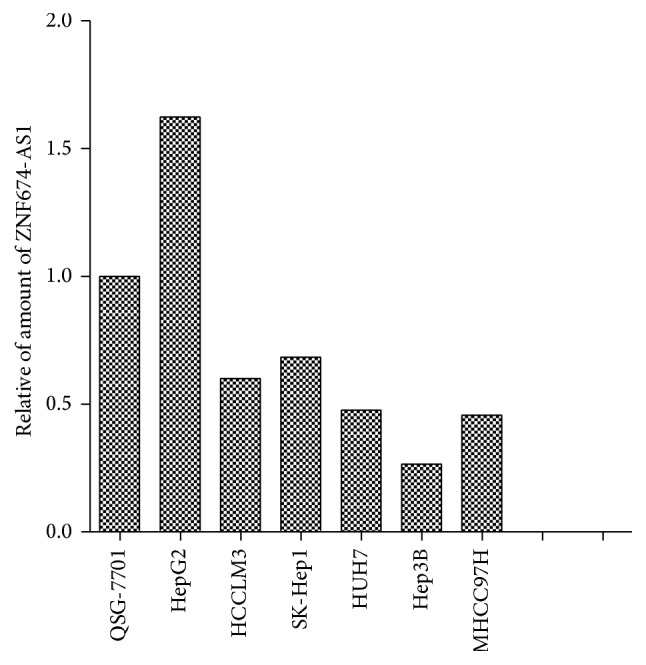
The ZNF674-AS1 expression level in HCC cell lines and a normal liver cell line QSG-7701.

Using qRT-PCR, we detected the expression levels of ZNF674-AS1 in HCC cell lines and tissues. We found that the expression level of ZNF674-AS1 in cancer tissues from patients with HCC was significantly lower than those in matched normal tissues (*p* < 0.01; Figure 1). Furthermore, the expression of ZNF674-AS1 was decreased in 72% (99/137) of HCC tissues compared with that in matched normal tissues (Figure 2). The expression of ZNF674-AS1 in five HCC cell lines (HCCLM3, SK-Hep1, HuH7, Hep3B, and MHCC97H) was significantly downregulated compared with that in the normal liver cell line QSG-7701 (Figure 3).

### 3.2. Relationship between the ZNF674-AS1 Levels in Cancer Tissues and Clinicopathological Factors in Patients with HCC

**Figure 4:**
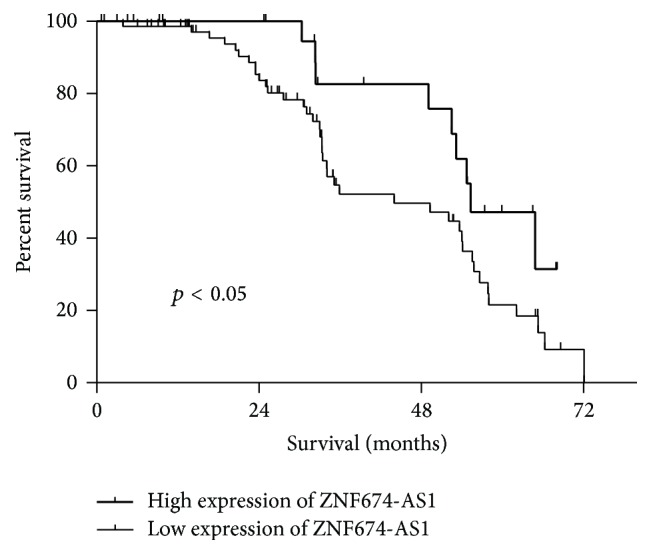
Kaplan–Meier curves estimating the 5-year recurrence-free survival rates according to the expression of the ZNF674-AS1 in patients with HCC who underwent hepatic resection.

Next, we explored whether the ZNF674-AS1 expression levels were associated with the clinicopathological factors of HCC. As shown in Table 1, the ZNF674-AS1 levels were associated with clinical stage (*p* = 0.039), histopathologic grade (*p* = 0.045), and cancer distal metastasis (*p* = 0.041). However, there was no significant correlation between ZNF674-AS1 expression and other clinicopathological features, such as age, gender, tumor diameter, hepatitis B, and liver cirrhosis. Finally, the assessment of overall survival in HCC patients revealed that a lower expression of ZNF674-AS1 was correlated with the adverse survival of patients with HCC (Figure 4).

## 4. Discussion

With the advances in high-resolution microarray and massively parallel sequencing technology, it has been well accepted that at least 90% of the human genome is actively transcribed into ncRNAs, while less than 2% of the genome sequences encode proteins [13, 15, 20]. Recent studies have highlighted that lncRNAs, larger than 200 nucleotides, are a new class of noncoding RNAs that might play critical roles in HCC progression [9, 12, 21].

ZNF674 antisense RNA 1 (ZNF674-AS1, NR_015378), located at Xp11.23, is a novel lncRNA that was identified from a lncRNA microarray analysis [10]. Zhu et al. demonstrated for the first time that ZNF674-AS1 was upregulated in 19 pairs of HCC samples compared with adjacent tumor samples [10]. However, in our study, we found that ZNF674-AS1 levels in 137 cancer tissues from patients with HCC were significantly lower than those in corresponding normal tissues. Further, the expression of ZNF674-AS1 in five HCC cell lines (HCCLM3, SK-Hep1, HuH7, Hep3B, and MHCC97H) was also significantly downregulated compared with the normal liver cell line QSG-7701. The differences between our study and Zhu et al.'s may be due to the number of HCC samples; 19 pairs of HCC samples are not sufficient to determine the expression level of ZNF674-AS1 in HCC.

To investigate the clinical value of ZNF674-AS1 in HCC diagnosis and prognosis, we investigated the correlation between the expression of ZNF674-AS1 and clinicopathological features of HCC. The statistical analysis indicated that low ZNF674-AS1 expression was significantly associated with clinical stage, histopathologic grading, and cancer distal metastasis. The Kaplan–Meier analysis showed that patients with low levels of ZNF674-AS1 expression tended to live a shorter life than those with high levels.

The present study has some limitations. Although ZNF674-AS1 may be a tumor suppressor gene, we have neither verified its role in vivo and in vitro nor found the underlying mechanism in the progression of HCC.

## 5. Conclusion

These results suggest that the aberrant expression of ZNF674-AS1 might be involved in the biological characteristics of HCC and might be a novel biomarker for predicting the free survival of HCC.

## Figures and Tables

**Table 1:** Clinicopathological correlation of ZNF674-AS1 expression in human hepatocellular carcinoma (HCC). AFP: Alpha Fetal Protein; PVTT: Portal Vein Tumor Thrombus; ^*∗*^ *p* < 0.05.

Parameters	Group	Total	ZNF674-AS1 expression		*p* value
			Low	High	
Gender	Male	118	85	33	0.88
	Female	19	14	5	

Age	<60	88	64	24	0.87
	≥60	49	35	14	

Hepatitis B	Absent	40	31	9	0.61
	Present	97	68	29	

Liver cirrhosis	Absent	40	31	9	0.38
	Present	97	68	29	

AFP	Negative	51	36	15	0.74
	Positive	86	63	23	

Tumor size	≤3	27	20	7	0.82
	>3	110	79	31	

Tumor number	Single	105	79	26	0.16
	Multiple	32	20	12	

PVTT	Absent	94	65	29	0.23
	Present	43	34	9	

Metastasis	Absent	121	84	37	0.04^*∗*^
	Present	16	15	1	

Clinical stage	I-II	102	69	33	0.039^*∗*^
	III-IV	35	30	5	

Histopathologic grading	Poorly	102	79	23	0.045^*∗*^
	Well + moderately	35	20	15	
